# Radiographic cortical thickness parameters as predictors of rotational alignment in proximal tibial shaft fractures: a cadaveric study

**DOI:** 10.1186/s12891-021-04452-w

**Published:** 2021-06-26

**Authors:** Alexander M. Keppler, Konstantin Küßner, Anna-Lena Schulze, Eduardo M. Suero, Carl Neuerburg, Maximilian Weigert, Christian Braun, Wolfgang Böcker, Christian Kammerlander, Christian Zeckey

**Affiliations:** 1 Department of General, Trauma and Reconstructive Surgery, University Hospital LMU Munich, Marchioninistraße 15, 81377, Munich, Germany; 2 Statistical Consulting Unit, StabLab, Department of Statistics, LMU Munich, Munich, Germany; 3 Institute of Legal and Forensic Medicine, University Hospital, LMU Munich, Munich, Germany; 4 Departement of Trauma and Orthopedic Surgery, RoMed Hospital, Pettenkoferstr.10, 83022 Rosenheim, Germany

**Keywords:** Rotational malalignment, Tibial shaft fracture, Cortical step sign, Diameter difference sign, Intramedullary nailing

## Abstract

**Aim:**

The treatment of tibial fractures with an intramedullary nail is an established procedure. However, torsional control remains challenging using intraoperatively diagnostic tools.

Radiographic tools such as the Cortical Step Sign (CSS) and the Diameter Difference Sign (DDS) may serve as tools for diagnosing a relevant malrotation. The aim of this study was to investigate the effect of torsional malalignment on CSS and DDS parameters and to construct a prognostic model to detect malalignment.

**Methods:**

A proximal tibial shaft fracture was set in human tibiae. Torsion was set stepwise from 0° to 30° in external and internal torsion. Images were obtained with a C-arm and transferred to a PC for measuring the medical cortical thickness (MCT), lateral cortical thickness (LCT), tibial diameter (TD) in AP and the anterior cortical thickness (ACT) as well as the posterior cortical thickness (PCT) and the transverse diameter (TD) of the proximal and the distal main fragment.

**Results:**

There were significant differences between the various degrees of torsion for each of the absolute values of the examined variables. The parameters with the highest correlation were TD, LCT and ACT. A model combining ACT, LCT, PCT and TD lateral was most suitable model in identifying torsional malalignment. The best prediction of clinically relevant torsional malalignment, namely 15°, was obtained with the TD and the ACT.

**Conclusion:**

This study shows that the CSS and DDS are useful tools for the intraoperative detection of torsional malalignment in proximal tibial shaft fractures and should be used to prevent maltorsion.

## Introduction

Tibial shaft fractures are one the most common long-bone fractures in adults [1–3]. Closed reduction and fixation with an intramedullary (IM) nail is the standard operative procedure for treating such fractures [4]. This minimally-invasive technique spares soft tissue damage compared to open reduction plate osteosynthesis and allows for immediate postoperative weight bearing [5].

Despite its advantages and widespread use, tibial nailing remains a technically challenging procedure. Postoperative, clinically-significant tibial maltorsion described as a torsional difference of 15 degrees or more between the main proximal and distal fragments, occurs in between 19 and 41% of cases [4, 6–9].

Tibial maltorsion may lead not only to cosmetic issues, but also to functional impairment of gait and stability [10–12]. In addition, tibial maltorsion causes increased and abnormally intraarticular contact forces, leading to a change in joint biomechanics and an increased rate of osteoarthritis in the knee and ankle [13, 14]. Therefore, significant postoperative tibial maltorsion is an indication for revision surgery.

Intraoperative tools for torsion control are limited and often not reproducible [15]. The gold standard for torsion control is a postoperative CT, which does not allow for intraoperative decision-making or corrections, therefore promoting additional revision procedures [16].

The Cortical Step Sign (CSS) and the Diameter Difference Sign (DDS) have been described as easy and feasible tools to identify potential maltorsion intraoperatively [17, 18]. In the case of torsional deformity, these radiographic signs show different thicknesses or diameters in the proximal and distal portions of the fracture fragments. These differences can easily be made visible intraoperatively using a mobile C-arm scanner. This sign could already be described for subtrochanteric femoral fractures where it was possible to identify intraoperative maltorsion [19]. Furthermore, the benefit of the CSS and DDS sign has already been demonstrated for medial shaft fractures in a cadaver model [20]. However, such evidence is lacking for proximal fractures of the tibia and the CSS and DDS are not further investigated in the literature so far.

The present study aims to evaluate for the first time the CSS and DDS in a proximal tibial shaft fracture cadaver model. We aimed to quantify the tibial CSS and DDS thresholds for clinically significant maltorsion in order to develop a model to accurately predict tibial maltorsion intraoperatively. Regarding the use of language, both terms such as torsion or rotation can be found in the literature. We deliberately use the term torsion here.

## Material and methods

The study was approved by the local Ethics Committee of the Medical Faculty (Nr. 18–184) and all procedures were followed in accordance with relevant guidelines. Nineteen fresh frozen human cadaveric tibia specimens were used. The tibia specimen were harvested by the Institute of Forensic Medicine of the Ludwig-Maximilians-University of Munich. There were four female donors and 15 male donors, with a median age of 61,.84 years (SD  ± 14,.4 years) and a median body mass index of 27.7 kg/m^2^ (SD  ± 4,.5 kg/m^2^). Table 1 shows the diameter of each specimen at the osteotomy site.

**Table 1:** Diameter of each Tibia specimen at the osteotomy site in the anteroposterior (AP) and lateral (Lat) radiographic views

	Diameter AP	Diameter Lat
1	31.3	42.2
2	34.5	35.9
3	32	34.3
4	35.7	36.7
5	25.1	29.2
6	31.1	36.6
7	23.5	30.7
8	30.3	40.4
9	28.7	37.7
10	30.2	36.7
11	28.5	43.5
12	37.1	35.8
13	29	34.5
14	31.4	34.7
15	29.7	37.3
16	27.2	31.6
17	29.2	38.4
18	31.3	37.1
19	25.7	31.6
**Mean**	30.1	36
**SD**	3.4	3.7

**Fig. 1:**
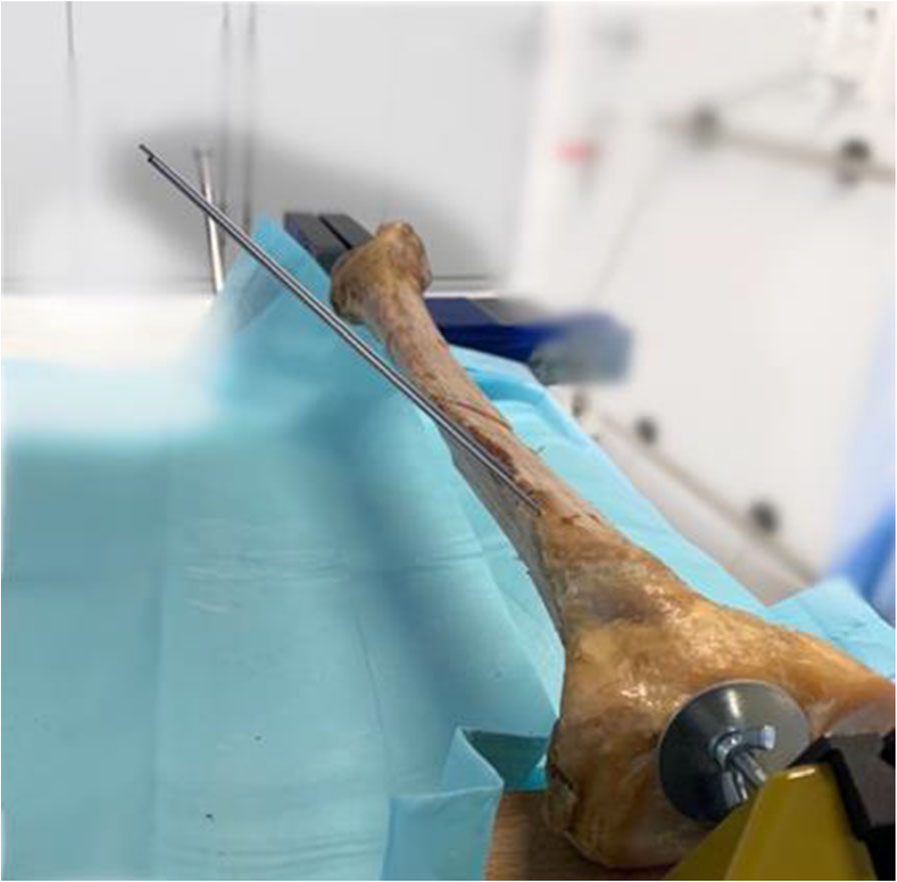
Experimental setup with K-wires to control torsional alignment of the tibial shaft

**Fig. 2:**
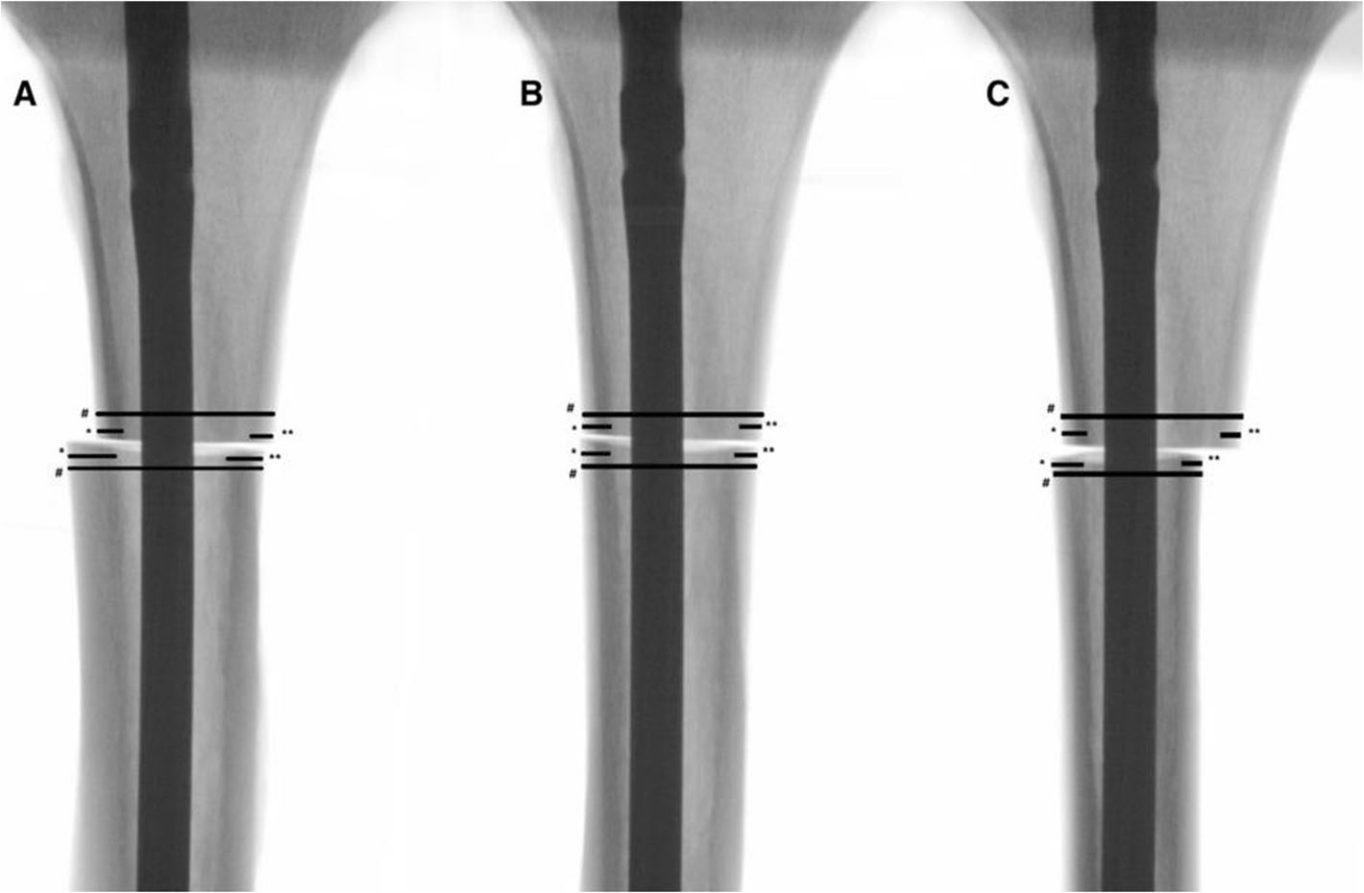
View of the different torsions in AP view. A: 20° external torsion B: 0° (neutral position) C: 20° internal torsion. # = Tibial shaft diameter (TD); * = Lateral cortical thickness (LCT), ** = Medial cortical thickness (MCT)

Every tibia was measured. Tibial length was defined as the distance from the tibial plateau to the tibial pilon. A transverse osteotomy was then performed at the junction of the proximal and the medial thirds to simulate a proximal tibial shaft fracture AO/OTA type 42-A3a. After the osteotomy a standard nailing procedure using an ETN® Tibial Nail (9 × 315 mm, DePuy Synthes, Umkirch, Germany) was performed. For torsion control, the proximal part of the tibia was fixed in a vise. A K-wire was inserted at the front edge of the tibia to control torsion. The torsion was then adjusted using a protractor, with the K-wire serving as a joystick. Using this system, stepwise torsional difference of the fragments was set at 5°, 10°, 15°, 20°, 25°, and 30° of external and internal torsion. The correct torsion was checked by two independent measurements using a protractor. Radiographic true anteroposterior (AP) and lateral views of the osteotomy site, as referenced by the posterior tibial crest, were obtained (Figs. 1 and 2) (Ziehm RF 3D, Ziehm Imaging, Nuremberg, Germany). The X-ray images were transferred to an image processing program (RadiAnt® DICOM Viewer) for taking measurements, which were scaled using the known diameter of the inserted nail. The CSS was evaluated measuring the medial cortical thickness (MCT), lateral cortical thickness (LCT), anterior cortical thickness (ACT) and posterior cortical thickness (PCT) of the tibia proximal and distal to the transverse osteotomy in a true anteroposterior (AP) and lateral view. A cortical thickness difference of 0.6 mm between the proximal and distal bone segments was defined as a positive CSS according to prior studies [19, 21]. For the analysis of the DDS, the transverse diameter of the femoral bone segment proximal and distal to the induced osteotomy was measured in true AP and lateral view (Tibial diameter [TD]). Analogous to the CSS, a difference of 0.6 mm of the proximal and distal femoral diameter was classified as a positive DDS.

### Statistics

We conducted correlation analyses between the absolute differences of the previously defined radiographic parameters and the magnitude of the torsional difference of the tibial fragments. These analyses were performed in analogy to preliminary work of our group on tibial mid-shaft fractures and subtrochanteric fractures [19, 20]. For each of the variables, an individual ANOVA regarding the thirteen levels of maltorsion as treatment was applied. Logistic regression models were then constructed to predict maltorsion greater than 15° as a function of the radiographic parameters. In order to provide guidance for the predictive power of specific radiographic parameters, individual regression models were used to calculate difference thresholds for probabilities between 0.5 und 0.9. Additionally, multiple logistic regression models using combinations of variables were estimated. To evaluate the predictive ability of the models, we conducted receiver operating characteristic (ROC) curve analyses and calculated positive predictive value (PPV), false discovery rate (FDR), sensitivity and specificity. The estimation of these performance measures was based on (5 × 5)-fold cross validation. For all conducted hypothesis tests, the significance level was set to α = 0.05. *P*-value adjustment due to multiple testing was accounted for.

## Results

### Analysis of plain radiographic parameters

**Fig. 3:**
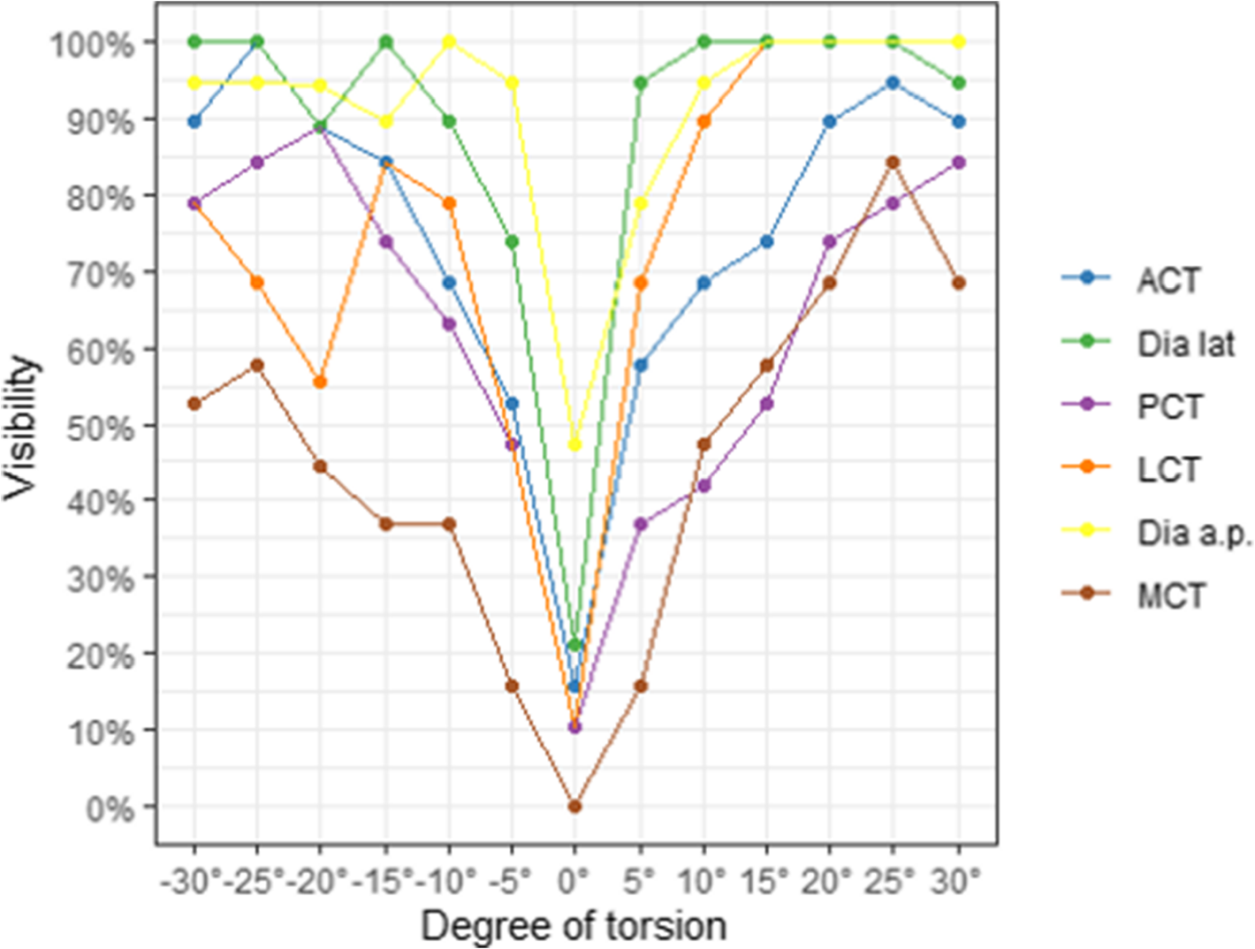
The influence of torsion on the visibility (0.6 mm) of individual parameter. The negative values reflect internal torsion, positive values reflect external torsion

In the analysis of the plain radiographs the AP view, LCT and TD were the most affected parameters in relation to tibial torsion (Table 2 and Fig. 3). There were significant differences for each of the absolute differences between the torsions (*p* <  0.001). In the lateral view, the ACT and TD parameters were more affected (Table 3 and Fig. 3).

**Table 2:** Absolute Differences in External Torsion, Anteroposterior View

	0°	5°	10°	15°	20°	25°	30°	p-Value
Medial Cortical Thickness								
MCT Mean	0.1 mm	0.4 mm	0.8 mm	1.04 mm	1.27 mm	1.61 mm	1.99 mm	< 0.001
MCT SD	0.13 mm	0.4 mm	0.76 mm	0.84 mm	0.85 mm	0.94 mm	1.42 mm	
MCT Visibility^1^	0%	15.79%	47.37%	57.89%	68.42%	84.21%	68.42%	
MCT Pos. Difference^1^	31.58%	36.84%	26.32%	15.79%	21.05%	21.05%	21.05%	
**Lateral Cortical Thickness**								
LCT Mean	0.15 mm	1.29 mm	2.27 mm	2.95 mm	3.64 mm	4.19 mm	4.4 mm	< 0.001
LCT SD	0.23 mm	1.11 mm	1.29 mm	1.51 mm	1.84 mm	1.97 mm	2.1 mm	
LCT Visibility^1^	10.53%	68.42%	89.47%	100%	100%	100%	100%	
LCT Pos. Difference^2^	47.37%	5.26%	0%	0%	0%	0%	0%	
**Diameter**								
Dia.ap Mean	0.81 mm	1.59 mm	2.52 mm	3.88 mm	5.06 mm	5.64 mm	6.02 mm	< 0.001
Dia.ap SD	0.83 mm	1.07 mm	1.35 mm	1.67 mm	1.87 mm	2.04 mm	2.61 mm	
Dia.ap Visibility^1^	47.37%	78.95%	94.74%	100%	100%	100%	100%	
Dia.ap Pos. Difference^2^	63.16%	31.58%	10.53%	5.26%	5.26%	5.26%	10.53%	

^1^ Visibility is given if the absolute difference exceeds 0.6 mm

^2^ A positive difference is equivalent to a greater proximal value compared to the distal value

**Table 3:** Absolute Differences in External Torsion, Lateral View

	0°	5°	10°	15°	20°	25°	30°	p-Value
Anterior Cortical Thickness								
ACT Mean	0.38 mm	0.89 mm	1.21 mm	1.62 mm	2.37 mm	2.95 mm	3.22 mm	< 0.001
ACT SD	0.94 mm	0.83 mm	0.88 mm	1.28 mm	1.51 mm	1.74 mm	1.81 mm	
ACT Visibility	15.79%	57.89%	68.42%	73.68%	89.47%	94.74%	89.47%	
ACT Pos. Difference	36.84%	63.16%	68.42%	78.95%	89.47%	89.47%	84.21%	
**Posterior Cortical Thickness**								
PCT Mean	0.17 mm	0.44 mm	0.71 mm	0.83 mm	1 mm	1.2 mm	1.42 mm	< 0.001
PCT SD	0.32 mm	0.38 mm	0.54 mm	0.67 mm	0.68 mm	0.65 mm	0.8 mm	
PCT Visibility	10.53%	36.84%	42.11%	52.63%	73.68%	78.95%	84.21%	
PCT Pos. Difference	47.37%	31.58%	42.11%	57.89%	73.68%	78.95%	78.95%	
**Diameter**								
Dia.lat Mean	0.41 mm	2.05 mm	3.47 mm	4.82 mm	6.31 mm	7.19 mm	7.32 mm	< 0.001
Dia.lat SD	0.52 mm	0.95 mm	1.36 mm	1.6 mm	1.97 mm	2.34 mm	3.19 mm	
Dia.lat Visibility	21.05%	94.74%	100%	100%	100%	100%	94.74%	
Dia.lat Pos. Difference	73.68%	94.74%	94.74%	89.47%	94.74%	94.74%	89.47%	

^1^ Visibility is given if the absolute difference exceeds 0.6 mm

^2^ A positive difference is equivalent to a greater proximal value compared to the distal value

External torsion in the AP view, in contrast to the lateral view of the tibia, leads to an increase in the diameter and thickness of the variables in the distal tibial fragment (Fig. 2).

When looking at internal torsion, the AP view showed changes in the LCT and TD values (Table 4), while the lateral view showed significant changes in each of the TD and ACT parameters. The distal tibial fragment showed an increase in thickness and diameter in all torsions analysed (Table 5). The various parameters and the influence of torsion on visibility at a threshold of 0.6 mm are shown in Fig. 3.

**Table 4:** Absolute Differences in Internal Torsion, Anteroposterior View

	0°	5°	10°	15°	20°	25°	30°	*p*-Value
Medial Cortical Thickness								
MCT Mean	0.1 mm	0.4 mm	0.72 mm	0.67 mm	0.84 mm	0.92 mm	1.03 mm	0.009
MCT SD	0.13 mm	0.5 mm	0.58 mm	0.79 mm	0.96 mm	0.97 mm	1.24 mm	
MCT Visibility	0%	15.79%	36.84%	36.84%	44.44%	57.89%	52.63%	
MCT Pos. Difference	31.58%	42.11%	57.89%	57.89%	55.56%	47.37%	52.63%	
**Lateral Cortical Thickness**								
LCT Mean	0.15 mm	0.85 mm	1.45 mm	1.52 mm	1.09 mm	1.61 mm	1.84 mm	< 0.001
LCT SD	0.23 mm	0.72 mm	0.86 mm	0.87 mm	0.9 mm	1.2 mm	1.28 mm	
LCT Visibility	10.53%	47.37%	78.95%	84.21%	55.56%	68.42%	78.95%	
LCT Pos. Difference	47.37%	57.89%	57.89%	63.16%	50%	52.63%	57.89%	
**Diameter A.P.**								
Dia.ap Mean	0.81 mm	2.12 mm	2.89 mm	3.11 mm	3.63 mm	3.49 mm	3.65 mm	< 0.001
Dia.ap SD	0.83 mm	1.29 mm	1.55 mm	1.74 mm	1.79 mm	2.01 mm	1.99 mm	
Dia.ap Visibility	47.37%	94.74%	100%	89.47%	94.44%	94.74%	94.74%	
Dia.ap Pos. Difference	63.16%	100%	94.74%	94.74%	94.44%	89.47%	89.47%	

^1^ Visibility is given if the absolute difference exceeds 0.6 mm

^2^ A positive difference is equivalent to a greater proximal value compared to the distal value

**Table 5:** Absolutes Differences in Internal Torsion, Lateral View

	0°	5°	10°	15°	20°	25°	30°	p-Value
Anterior Cortical Thickness								
ACT Mean	0.38 mm	0.75 mm	1.25 mm	1.89 mm	2.13 mm	2.16 mm	2.36 mm	< 0.001
ACT SD	0.94 mm	0.54 mm	0.87 mm	0.94 mm	1.04 mm	1.14 mm	1.41 mm	
ACT Visibility	15.79%	52.63%	68.42%	84.21%	88.89%	100%	89.47%	
ACT Pos. Difference	36.84%	10.53%	15.79%	15.79%	5.56%	10.53%	21.05%	
**Posterior Cortical Thickness**								
PCT Mean	0.17 mm	0.78 mm	1.08 mm	1.65 mm	1.85 mm	1.85 mm	1.78 mm	< 0.001
PCT SD	0.32 mm	0.74 mm	0.98 mm	1.07 mm	1.14 mm	1.35 mm	1.04 mm	
PCT Visibility	10.53%	47.37%	63.16%	73.68%	88.89%	84.21%	78.95%	
PCT Pos. Difference	47.37%	21.05%	15.79%	10.53%	11.11%	5.26%	15.79%	
**Diameter Lateral**								
Dia.lat Mean	0.41 mm	1.16 mm	2.15 mm	2.85 mm	3.19 mm	3.18 mm	3.42 mm	< 0.001
Dia.lat SD	0.52 mm	0.69 mm	1.17 mm	1.34 mm	1.79 mm	1.79 mm	1.86 mm	
Dia.lat Visibility	21.05%	73.68%	89.47%	100%	88.89%	100%	100%	
Dia.lat Pos. Difference	73.68%	10.53%	5.26%	5.26%	5.56%	5.26%	5.26%	

^1^ Visibility is given if the absolute difference exceeds 0.6 mm

^2^ A positive difference is equivalent to a greater proximal value compared to the distal value

### Correlation between plain radiographic parameters and Tibial Maltorsion

**Fig. 4:**
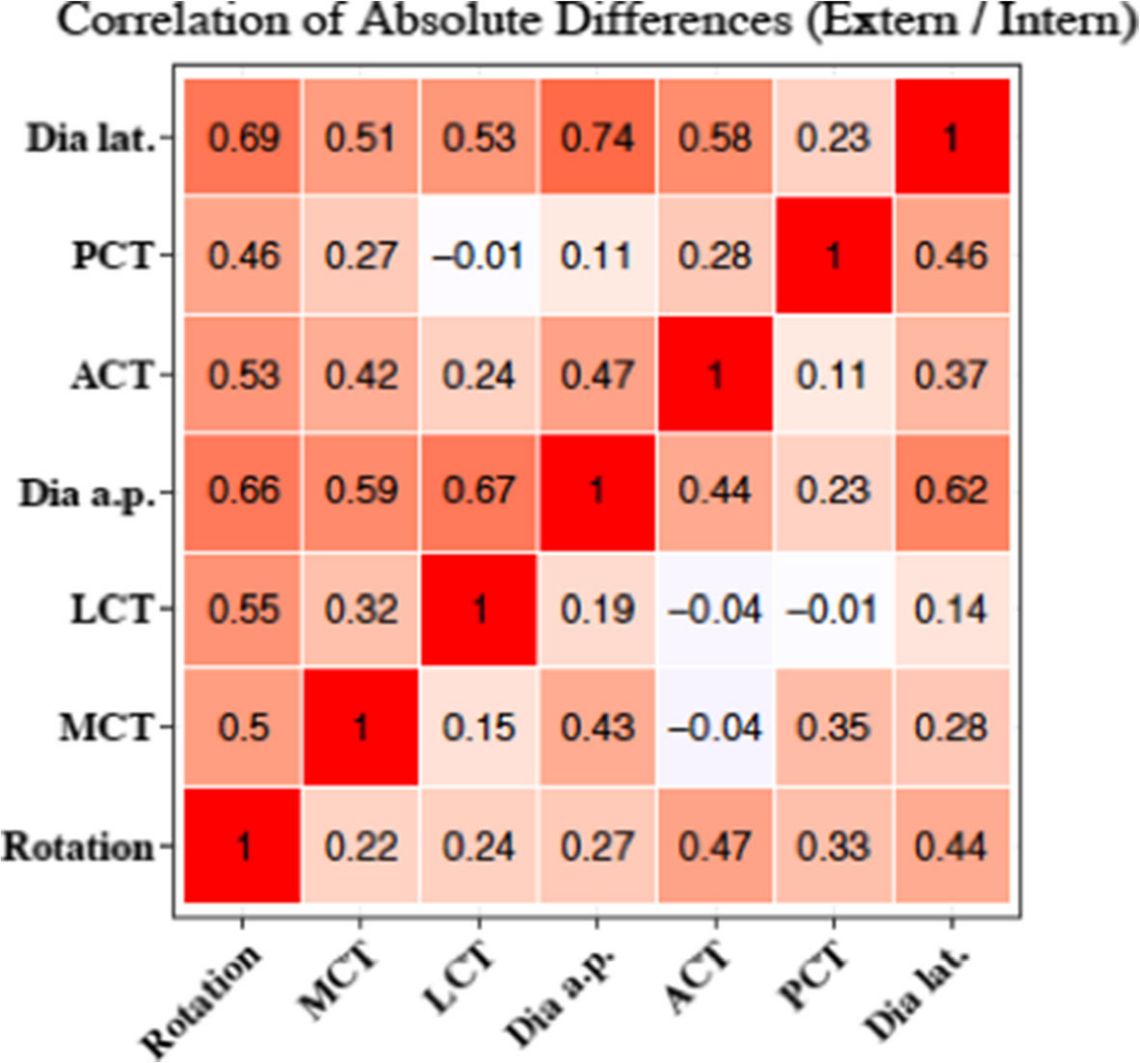
Correlation of absolute radiographic differences and torsional alignment. Upper triangle: External torsion. Lower triangle: Internal torsion

The radiological parameters TD in the lateral view, TD in the anteroposterior view and the LCT each showed the highest correlation for external torsion (TD lat: 0.69; TD ap: 0.66; LCT: 0.55). The TD value was the most strongly associated variable for external torsion. An individual comparison of the variables showed the highest correlation for the variables TD lat and TD ap with a value of 0.74 for external torsion (Fig. 4).

### Probability of Maltorsion by measurement threshold

Using logistic regression models, threshold values for the torsion variables were found at the clinically relevant limit of 15°. When comparing the MCT and the LCT, it was shown that even small changes in the MCT serve to identify a clinically relevant torsional malposition. A probability level of 0.8 was found for a difference of 2.63 mm, showing a PPV of 0.77 and a specificity of 0.97.

In contrast, in the ap view, a threshold of 6.27 mm was required to achieve a probability of 0.8 and a specificity of 0.99.

Overall, the lateral view showed more discrete changes in the PCT than in the ACT values to detect torsional malalignment: at a probability level of 0.8, a difference of 2.94 mm was required in the PCT with a PPV of 0.53 and a specificity of 0.97, compared to the same probability level for the ACT, a change of 3.92 mm was required, resulting in a PPV of 0.78 and a specificity of 0.96.

For the tibial diameter, a threshold of 6.85 mm was required for a probability level of 0.8. This leads to a PPV of 0.91 and a high specificity of 0.98. The detailed results are shown in Table 6.

**Table 6:** Probability of Tibial maltorsion at each measurement threshold for each of the radiographic variables studied

Probability	Threshold	PPV	FDR	Sensitivity	Specificity
Medial Cortical Thickness	(*p* < 0.001)				
0.5	1.06	0.63	0.37	0.46	0.82
0.6	1.52	0.60	0.40	0.33	0.89
0.7	2.02	0.68	0.32	0.24	0.94
0.8	2.63	0.77	0.23	0.15	0.97
0.9	3.55	1.00	0.00	0.04	1.00
**Lateral Cortical Thickness**	**(p < 0.001)**				
0.5	2.42	0.55	0.45	0.49	0.77
0.6	3.30	0.55	0.45	0.35	0.86
0.7	4.25	0.63	0.37	0.24	0.94
0.8	5.42	0.91	0.09	0.13	0.99
0.9	7.17	1.00	0.00	0.04	1.00
**Diameter A.P.**	**(p < 0.001)**				
0.5	3.72	0.47	0.53	0.61	0.76
0.6	4.47	0.60	0.40	0.51	0.87
0.7	5.28	0.65	0.35	0.40	0.93
0.8	6.27	0.94	0.06	0.26	0.99
0.9	7.76	1.00	0.00	0.06	1.00
**Anterior Cortical Thickness**	**(p < 0.001)**				
0.5	1.92	0.49	0.51	0.63	0.76
0.6	2.39	0.66	0.34	0.49	0.89
0.7	2.90	0.86	0.14	0.36	0.94
0.8	3.52	0.75	0.25	0.24	0.96
0.9	4.45	0.91	0.09	0.14	0.99
**Posterior Cortical Thickness**	**(p < 0.001)**				
0.5	1.30	0.59	0.41	0.51	0.75
0.6	1.78	0.60	0.40	0.34	0.85
0.7	2.30	0.75	0.25	0.19	0.94
0.8	2.94	0.48	0.52	0.06	0.97
0.9	3.90	0.95	0.05	0.04	1.00
**Diameter Lateral**	**(p < 0.001)**				
0.5	3.91	0.42	0.58	0.62	0.77
0.6	4.77	0.52	0.48	0.52	0.88
0.7	5.71	0.75	0.25	0.43	0.95
0.8	6.85	0.93	0.07	0.30	0.99
0.9	8.57	1.00	0.00	0.13	1.00

### ROC analysis of multiple logistic regression models

**Fig. 5:**
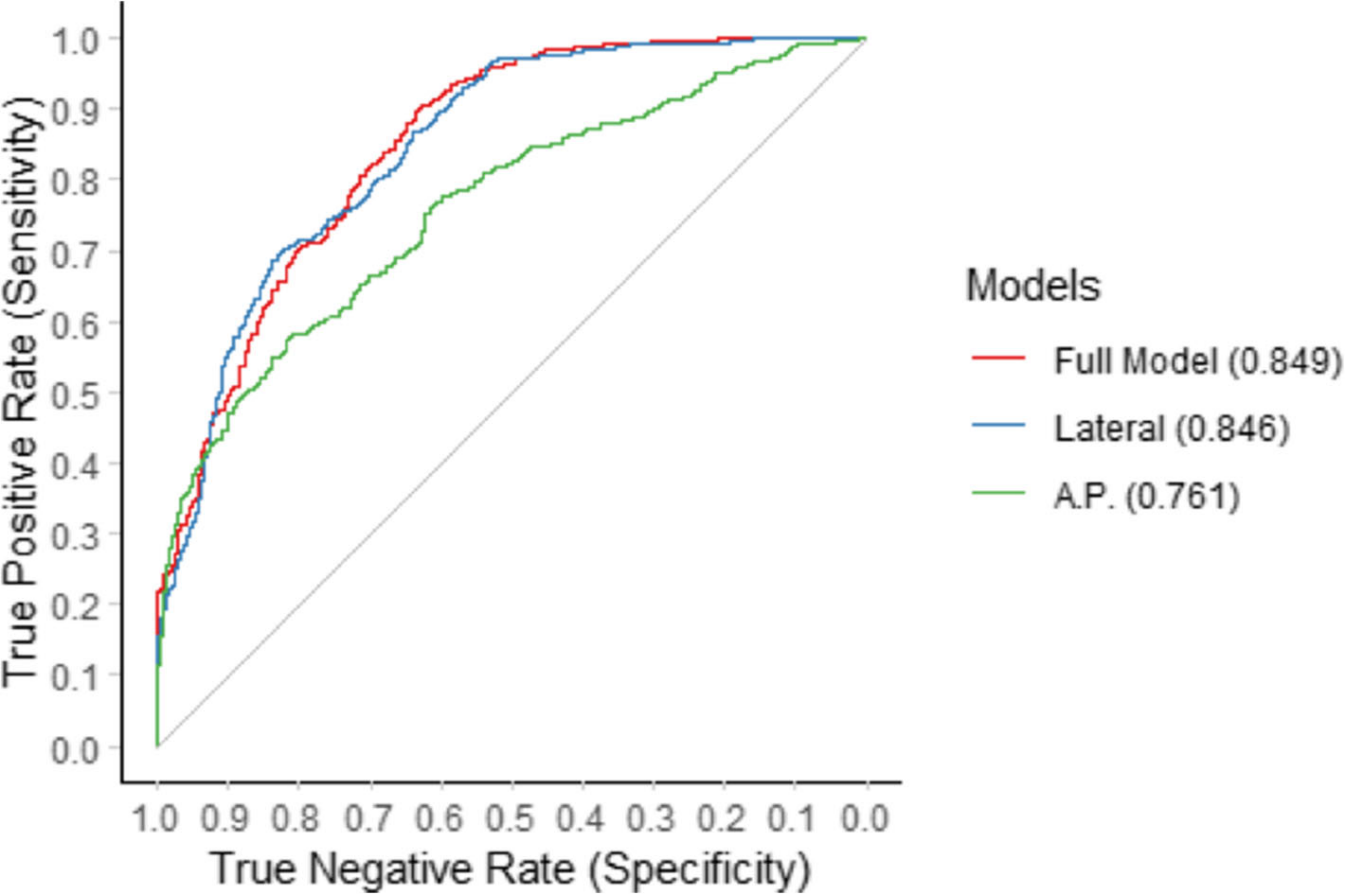
Comparison of receiver operating characteristic (ROC) curves of the univariate models of tibial maltorsion as a function of radiographic cortical and diameter parameters

**Fig. 6:**
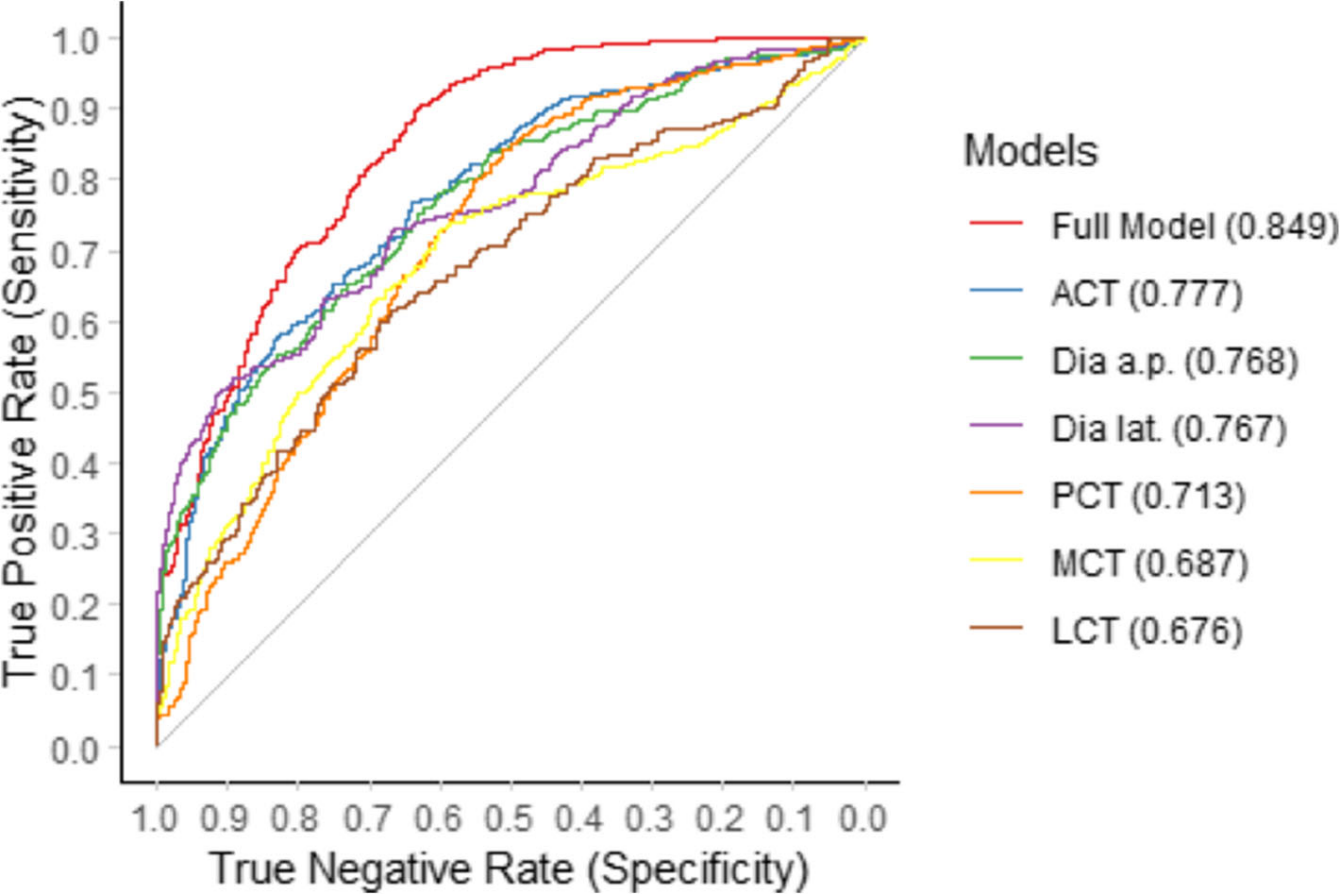
Receiver operating characteristic (ROC) curves of the models of tibial maltorsion as a function of the combination of variables in the anterior posterior or lateral views demonstrating improved sensitivity and specificity

To further analyse the respective thresholds and the influence of the models on torsion on specificity and sensitivity, we combined the AP and lateral view variables to perform a ROC curve analyses. The ROC curves clearly showed benefit from the isolated variables described above and improved prediction when the values were combined. When looking at the individual values of the views alone, the combination of variables in the lateral view showed better accuracy than the variables in the AP view. See Figs. 5 and 6 for details of the two models.

## Discussion

To the best of our knowledge this is the first study quantifying and describing the usability of the cortical step and diameter difference signs to detect torsional malalignment in the proximal tibia shaft. We were able to demonstrate the reproducibility and accuracy of the cortical step sign and the diameter difference sign under experimental conditions. We showed that external torsion leads to increased thickness in the cortical parameters of the distal fragments, while internal torsion leads to decreased thickness. Furthermore, we showed that the lateral view was particularly useful in predicting tibial maltorsion.

Tibial maltorsion is a dreaded complication after intramedullary stabilisation of tibial shaft fractures. The degree of maltorsion which is clinically relevant is not precisely defined. Most studies assume 10°, with a range of 5–15° [7]. In this study, we chose a value of 15° for tibial maltorsion, as it is often the reason for a surgical revision in the clinic. Studies have shown an increased incidence of osteoarthritis, as well as an increased risk of pseudarthrosis in people with tibial maltorsion of more than 10 degrees and still functional limitations after one year [22–24]. While an indication for revision surgery is possible after postoperative identification of a clinically significant tibial maltorsion, represents additional risks for the patient and an increased financial burden for the health care system. Proximal tibial shaft fractures in particular appear to be challenging and therefore require good surgical management and meticulous reposition [25].

Although several algorithms for detecting torsional differences have been described, the incidence of clinically relevant torsional malalignment remains unacceptably high [26]. In the case of suspected torsional malalignment, CT of the lower extremities is the gold standard for detection and quantification of tibial maltorsion [7].

Despite its high clinical relevance, only a few attempts have been made to intraoperatively detect and correct tibial maltorsion [15, 27]. Inci et al. described a method that could enable a correct adjustment of the tibial torsion with the help of an external target device. This method offers the advantage of not increasing radiation exposure, but requires special instruments and experience [28]. Another method described in literature is the insertion of two K-wires into the tibia in order to achieve optical torsional control [29]. Thus, a practical, accurate, valid, consistent and reproducible method/tool is required. Ideally, this method should be easy to produce and require no major technical equipment. Although there are approaches to torsion control through the possibilities of intraoperative navigation, these are technically complex and associated with immense costs.

On the other hand, numerous methods have been described for the postoperative assessment of maltorsion. CT imaging usually plays a decisive and important role in this context [30]. However, it entails increased radiation burden and any identified maltorsion may only be corrected with additional surgery. Therefore, reliable intraoperative detection offers decisive advantages. In 1998 Krettek et al. described for the first time in a technical note the applicability of CSS for tibial shaft fractures [18]. However, clinical investigation and studies on the feasibility of this sign for tibial fractures are still lacking. The authors’ research group has already demonstrated the usefulness of the CSS and DDS parameters for tibial mid-shaft fractures [20]. This work now shows comparable results for the proximal tibial shaft.

CSS and DDS have been better studied in the setting of femoral fractures, and several authors have reported on its clinical usefulness [19, 31, 32]. Fang et al. could show in a study on the femur that especially the lateral view on the lemur is used for interpretation [21]. This is consistent with our results where DDS and CSS are also more visible in the lateral view.

There are some limitations to our present study. We only examined one osteotomy site (proximal tibial shaft fractures) and fracture type. It would be interesting to see how the parameters behave depending on the height of the osteotomy or the type of fracture. This could be investigated in further studies. However, our experimental setup has the advantage of serving as a highly controlled model, in which the effect of torsion on the radiographic parameters can be accurately quantified. It offers the possibility to investigate the characteristics under controlled conditions without the aid of a CT scan or other technical aids. This opens up the possibility of being able to use this technology easily at any time, even without complex infrastructure or high investments.

## Conclusion

We have quantified the cortical step sign and diameter difference sign at various degrees of tibial maltorsion after intramedullary nailing for proximal shaft fractures. In addition, we have constructed a model to accurately predict tibial maltorsion based on intraoperative radiographic parameters. This study shows feasibility and applicability of the cortical step sign and the diameter difference sign in tibial shaft fractures. The CSS and DDS could be promising tools for detecting torsional malalignment in proximal tibial fractures, potentially decreasing the incidence of clinically relevant maltorsion and reducing the need for additional revision procedures.

## Data Availability

The datasets used during the current study available from the corresponding author on reasonable request.
